# Deep learning for accurate B-line detection and localization in lung ultrasound imaging

**DOI:** 10.3389/frai.2025.1560523

**Published:** 2025-04-22

**Authors:** Nixson Okila, Andrew Katumba, Joyce Nakatumba-Nabende, Cosmas Mwikirize, Sudi Murindanyi, Jonathan Serugunda, Samuel Bugeza, Anthony Oriekot, Juliet Bossa, Eva Nabawanuka

**Affiliations:** ^1^Department of Computer Science, Makerere University, Kampala, Uganda; ^2^Department of Electrical and Computer Engineering, Makerere University, Kampala, Uganda; ^3^Department of Radiology Makerere University Hospital, Makerere University, Kampala, Uganda; ^4^Mulago Specialized Women and Neonatal Hospital, Kampala, Uganda; ^5^Mulago National Referral Hospital, Kampala, Uganda

**Keywords:** deep learning, lung ultrasound, B-line artifact, localization, YOLO

## Abstract

**Introduction:**

Lung ultrasound (LUS) has become an essential imaging modality for assessing various pulmonary conditions, including the presence of B-line artifacts. These artifacts are commonly associated with conditions such as increased extravascular lung water, decompensated heart failure, dialysis-related chronic kidney disease, interstitial lung disease, and COVID-19 pneumonia. Accurate detection of the B-line in LUS images is crucial for effective diagnosis and treatment. However, interpreting LUS is often subject to observer variability, requiring significant expertise and posing challenges in resource-limited settings with few trained professionals.

**Methods:**

To address these limitations, deep learning models have been developed for automated B-line detection and localization. This study introduces YOLOv5-PBB and YOLOv8-PBB, two modified models based on YOLOv5 and YOLOv8, respectively, designed for precise and interpretable B-line localization using polygonal bounding boxes (PBBs). YOLOv5-PBB was enhanced by modifying the detection head, loss function, non-maximum suppression, and data loader to enable PBB localization. YOLOv8-PBB was customized to convert segmentation masks into polygonal representations, displaying only boundaries while removing the masks. Additionally, an image preprocessing technique was incorporated into the models to enhance LUS image quality. The models were trained on a diverse dataset from a publicly available repository and Ugandan health facilities.

**Results:**

Experimental results showed that YOLOv8-PBB achieved the highest precision (0.947), recall (0.926), and mean average precision (0.957). YOLOv5-PBB, while slightly lower in performance (precision: 0.931, recall: 0.918, mAP: 0.936), had advantages in model size (14 MB vs. 21 MB) and average inference time (33.1 ms vs. 47.7 ms), making it more suitable for real-time applications in low-resource settings.

**Discussion:**

The integration of these models into a mobile LUS screening tool provides a promising solution for B-line localization in resource-limited settings, where accessibility to trained professionals may be scarce. The YOLOv5-PBB and YOLOv8-PBB models offer high performance while addressing challenges related to inference speed and model size, making them ideal candidates for mobile deployment in such environments.

## 1 Introduction

**Figure 1:**
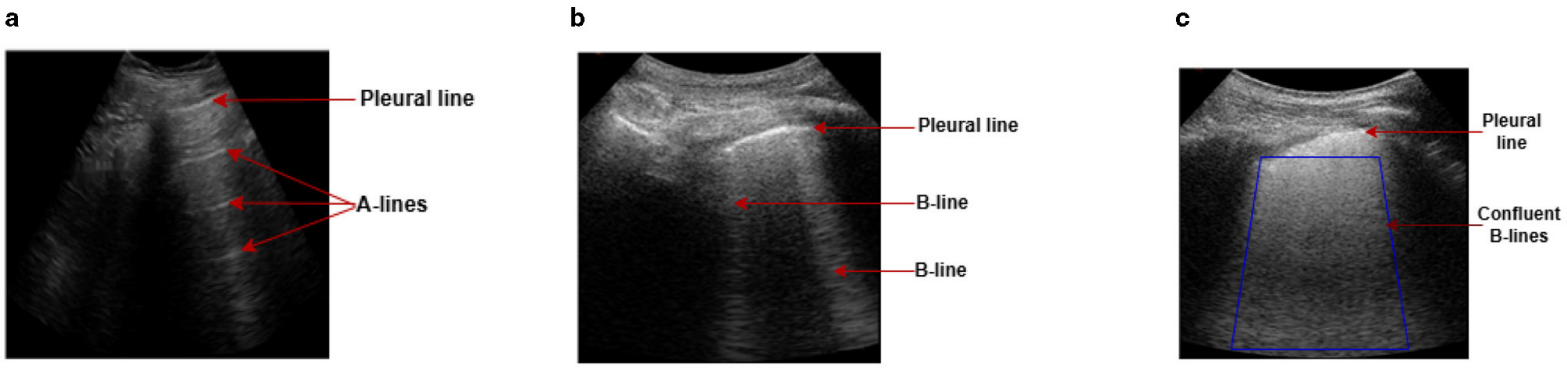
Examples of LUS images captured during data collection, illustrating A-line and B-line LUS imaging artifacts. **(a)** In a healthy lung, ultrasound waves are reflected at the pleura, resulting in horizontal reverberation artifacts known as A-lines. **(b)** As fluid volume increases, vertical artifacts (B-lines) become common, characterized by bright lines that originate from the pleural line. **(c)** In severe cases, B-lines may merge together and become confluent, spanning the entire intercostal space.

Lung ultrasound (LUS) has emerged as a vital imaging tool in the field of pulmonary and critical care medicine due to its non-invasiveness, portability, and safety (Zhou et al., 2024). Unlike traditional imaging modalities such as X-rays or computed tomography scans, LUS can be performed at the point of care, providing rapid diagnostic insight without needing radiation exposure (Yang et al., 2023). This advantage is particularly relevant in the context of respiratory diseases, where timely and accurate diagnosis can significantly impact patient outcomes. The basis of LUS is the relative amounts of fluid and air (the air/fluid ratio) in the lung, which defines the characteristics of the image seen in LUS and may vary depending on the lung's level of aeration. A healthy lung would mostly be filled with air, and because of the high mismatch in acoustic impedance between the air and the tissue, the ultrasound waves are reflected to the transducer during scanning. These reflected waves bounce back and forth between the pleural line and the transducer multiple times before they are finally detected, creating a series of horizontal lines on the LUS image at regular intervals. These horizontal lines, commonly known as A-lines, are seen parallel to the pleural line (Demi et al., 2023) (Figure 1a). When lung health deteriorates, air volume reduces, allowing less attenuating substances such as fluid or other biological materials to accumulate. This allows the LUS field to penetrate deeper into the lung, leading to greater visibility of vertical artifacts like B-lines (Soldati et al., 2020). Recent research suggests that B-lines are generated by the internal reflection of LUS waves within semi-aerated alveoli, which act as acoustic traps (Demi et al., 2020). The B-lines normally appear as hyper-echoic discrete vertical artifacts that originate from a point along the pleura-line and lie perpendicular to the latter (Demi et al., 2023) (Figure 1b). In a severe diseased state, the B-lines may merge to form confluent B-lines, spanning the entire intercostal space (Fischer et al., 2022) (Figure 1c).

In clinical practice, B-lines are among the most important artifacts in LUS for detecting and evaluating the severity of pulmonary congestion in patients with conditions such as pulmonary edema, decompensated heart failure, kidney disease, pneumonia, interstitial lung disease, or infections caused by SARS-CoV-2 such as coronavirus disease 2019 (COVID-19) (Demi et al., 2023; Luna et al., 2022). Precise localization of B-lines in LUS images allows clinicians to differentiate between various lung pathologies, understand the extent of lung involvement, and make informed decisions regarding patient management (Piculjan et al., 2020). However, unlike ultrasound imaging of other organs and tissues, LUS does not offer a direct anatomical representation of the lung parenchyma in its images (Soldati et al., 2019). This is because the high air content in the lung obstructs ultrasound waves from penetrating the parenchyma (Demi et al., 2023). Instead, various scattering phenomena are produced and shaped by the size and distribution of air-filled compartments, such as alveoli, which prevent conventional B-mode anatomical imaging (Gino et al., 2020). As a result, LUS relies on the observation and interpretation of imaging artifacts, such as A-lines and B-lines. However, manual interpretation of LUS imaging artifacts is subject to intra- and inter-observer variability and requires substantial expertise (Lucassen et al., 2023). This variability presents a significant challenge, particularly in resource-limited settings where trained professionals may be unavailable. Automating the detection and localization of B-lines through advanced computational methods, such as deep learning, presents an opportunity to standardize LUS interpretation and extend its utility in diverse clinical environments.

Deep learning (DL), a subset of artificial intelligence, has shown remarkable success in image analysis tasks across various domains, including medical imaging (Zhao et al., 2022). It has recently drawn much attention in LUS imaging, particularly for tasks such as image classification, artifact detection, and segmentation (Howell et al., 2024). The most commonly used deep learning techniques for vision-related tasks rely on convolutional neural networks (CNNs) because of their powerful ability to extract features from visual data. The CNN architecture comprises convolutional layers that automatically learn and extract hierarchical features from input data, allowing it to identify complex patterns in images efficiently. Despite the potential of DL in automating LUS interpretation, several challenges remain. The heterogeneous nature of ultrasound data, which varies based on patient demographics, equipment settings, and scanning protocols, presents a significant hurdle for model generalization. Additionally, the complex appearance of B-lines, which can be affected by factors such as noise and the presence of other artifacts (e.g., A-lines, pleural irregularities), requires models that are robust and capable of distinguishing between different types of echogenic patterns. Addressing these challenges is essential for developing deep learning solutions that are reliable and widely applicable.

By leveraging large annotated datasets, DL models, such as You Only Look Once (YOLO), can be trained to identify and localize B-lines in LUS, potentially replicating or surpassing human-level performance. YOLO is a family of real-time object detection models renowned for their efficiency and speed in identifying and localizing objects within images. Unlike traditional object detection algorithms that rely on region-based approaches, YOLO redefines the problem by treating object detection as a single regression task. It predicts both class probabilities and bounding box coordinates for objects in an image simultaneously, making it a highly efficient framework for real-world applications requiring speed and precision (Joseph and Ali, 2017). The evolution of YOLO, from its early versions to the more advanced YOLOv5 and YOLOv8, showcases remarkable improvements in precision, scalability, and functionality (Jocher et al., 2023). YOLOv5 introduced optimizations such as better anchor box mechanisms and lighter architectures, while YOLOv8 integrates advanced segmentation capabilities, enabling pixel-wise predictions alongside bounding box detections (Hussain, 2024). These enhancements make YOLO adaptable to various tasks, including object detection, segmentation, and localization in medical imaging.

Recent studies have explored various methods for LUS image analysis, including the localization and segmentation of B-lines (Cristiana et al., 2020; Van Sloun and Demi, 2020; Hamideh et al., 2021; Sourabh et al., 2018; Subhankar et al., 2020; Zhao et al., 2022; Howell et al., 2024). However, the models proposed in Cristiana et al. (2020) and Sourabh et al. (2018) face challenges in accurately capturing the shapes of B-line artifacts, while those in Van Sloun and Demi (2020), Hamideh et al. (2021), Subhankar et al. (2020), Zhao et al. (2022), Howell et al. (2024), and Lucassen et al. (2023) fail to preserve the fine-grained details of the artifacts, diminishing their interpretability. Additionally, none of these models have been incorporated into a mobile LUS screening tool for deployment in resource-constrained settings. To address these limitations, there is need for a model that can accurately capture the shapes and fine-grained details of B-line artifacts, while also being integrated into a mobile LUS screening tool for use in resource-limited settings. This study aims to bridge these gaps by developing a more precise and interpretable model for B-line artifact localization, ultimately improving the accessibility and effectiveness of LUS analysis in underserved environments.

This article explores the adaptation of YOLOv5 and YOLOv8 object detection models for precise localization of B-line artifacts in lung ultrasound (LUS) using polygonal bounding boxes (PBBs). YOLOv5, an anchor-based detection model, and YOLOv8, an anchor-free variant with improved feature extraction and segmentation capabilities, were both employed to assess their their accuracy, inference speed, and computational complexity in localizing B-line artifacts. To achieve PBB localization in YOLOv5, modifications were made to the detection head, non-maximum suppression function, loss function, and data loader. Meanwhile, in the YOLOv8 segmentation model, the post-processing was customized to fit polygons onto the segmented mask while removing the mask to preserve the fine-grained details of B-line artifacts. Additionally, a tailored image preprocessing technique, developed using the OpenCV and Keras-OCR libraries, was integrated into the models to enhance LUS image quality by extracting the region of interest (RoI) and eliminating ultrasound text labels. The method first applies Otsu's thresholding to differentiate foreground objects from the background, followed by morphological operations to refine region boundaries. OpenCV's contour detection algorithm is then used to identify and extract the RoI. Subsequently, the Keras-OCR identifies ultrasound text labels and applies a mask to highlight them. Furthermore, the models have been incorporated into a mobile LUS screening tool for deployment in resource-limited areas. The main contributions of this study can be summarized as follows.

1. We improved the quality of LUS images by implementing a customized image preprocessing technique that uses OpenCV and Keras-OCR libraries to extract the region of interest (RoI) from the image and remove ultrasound text labels.
2. We modified the YOLOv5 object detection model for PBB localization of B-line artifacts by adjusting the detection head, non-maximum suppression function, loss function, and data loader within its architecture.
3. We adapted the YOLOv8 segmentation model by customizing its post-processing function to fit polygons onto the segmented mask, while preserving the fine-grained details of the B-line artifacts.
4. We integrated the model, featuring a smaller size and faster inference, into a mobile LUS screening tool for deployment in resource-constrained settings.

The rest of the paper is organized as follows. Section 2 reviews related works, while Section 3 outlines the methods used in the study. The results are presented in Section 4. Section 5 illustrates the integration of the model into the LUS system. Section 6 provides a discussion, and Section 7 concludes the paper.

## 2 Related work

To date, several studies have investigated the detection of B-line artifacts in LUS using DL with different techniques, including gradient-based class activation mapping (Grad-CAM), rectangular bounding box (RBB), single-point and segmentation. For example, Cristiana et al. (2020) intended to quantify the evaluation of B-lines in LUS. They focused on the binary classification of the presence or absence of B-lines with a sensitivity of 93% and specificity of 96%. In addition, they attempted to measure the severity of B-lines on a scale of 0–4. Although this study showed potential, it is not transparent enough to describe how the model makes predictions, which reduces the clinicians' trust toward the model for computer-assisted diagnosis (Choy et al., 2018). Differently, Van Sloun and Demi (2020) proposed a DL model for automatic B-line detection in LUS image frames, using Grad-CAM, with the possibility of being implemented in real-time at 276 frames per second. Although Grad-CAM provides visual insights into the model's decision-making process (Talaat et al., 2024), its precision in delineating B-line boundaries is limited, reducing its effectiveness for fine-grained localization. Additionally, in LUS images containing multiple closely spaced B-lines, the generated heatmaps may overlap, making it challenging to interpret the B-line patterns (Tjoa and Guan, 2020). Similarly, Hamideh et al. (2021) integrated long short-term memory (LSTM) network and temporal attention mechanism to localize B-lines in LUS videos using spatial attention map (SAP). The model could localize the most salient B-line regions both spatially and temporally with a correlation coefficient of 0.67 and an IoU of 69.7%, respectively. However, the SAP approach highlights B-line regions broadly rather than pinpointing the exact boundaries of the artifact, making it difficult to understand the semantic context of the artifacts. In addition, the complex design of LSTM frequently strains the hardware (Yang et al., 2023). In 2018, Sourabh et al. (2018) presented a single-shot object detector for localizing pleural line, A-line artifacts, B-line artifacts, pleural effusion, and lung consolidations in swine lung ultrasound images. The authors implemented the RBB localization technique, and their model attained at least 85% in sensitivity and specificity for all features, except B-line sensitivity. While this strategy of RBB detection is simple and shows promise, it does not capture the exact shape and orientation of B-line artifacts. In most cases, some parts of the object might be left out, or irrelevant background might be included, leading to unnecessary computational overhead or false positives/negatives in detection tasks. In addition, since all data were collected from animals with induced pathology, further research is needed to see whether the model can be generalized to human LUS videos.

Other researchers have presented a segmentation approach for B-line detection in LUS. In 2020, Subhankar et al. proposed a model based on a spatial transformers network to segment healthy lung features and COVID-19 imaging biomarkers in LUS with an accuracy of 96%, utilizing this to develop a preliminary approach to severity assessment (Subhankar et al., 2020). Zhao et al. (2022) trained deep neural networks (DNNs) on simulated LUS images to segment B-line artifacts and the (DNNs) attained mean dice similarity coefficient (DSC) of 0.45. Relatedly, Howell et al. (2024) presented a lightweight U-Net model to segment the ribs, pleural line, A-lines, discrete and confluent B-lines in B-mode images of a COVID-19 lung tissue-mimicking phantom with mean DSC of 0.74. While these segmentation techniques in Subhankar et al. (2020), Zhao et al. (2022), and Howell et al. (2024) allow precise delineation of features, it is challenging to understand the meaning or significance of specific regions within the mask without additional context. Alternatively, Lucassen et al. (2023) proposed a segmentation network, comprising an ensemble of EfficientNet-B0 and U-Net decoder for single-point localization of B-lines. The network was trained using single-point annotations located where B-lines originate from the pleura. Although this technique can predict the landmark locations of discrete B-lines as single points with an F1-score of 0.65, the prediction of B-line origins can coalesce for merged B-lines or discrete B-lines in close proximity, making imprecise predictions of B-lines origins on the pleura.

In summary, various deep learning approaches have been investigated for detecting and localizing B-line artifacts in LUS, each with distinct advantages and limitations. The classification model in Cristiana et al. (2020) achieves high sensitivity and specificity but lacks interpretability, which may reduce clinical trust. Methods like the Grad-CAM-based approach in Van Sloun and Demi (2020) and the SAP-based technique in Hamideh et al. (2021) provide insights into model predictions and localization but do not capture the precise boundaries of B-lines. The RBB method in Sourabh et al. (2018) is simple but limited in accurately representing the shape, orientation, and generalizability of artifacts to human data. Segmentation models proposed in Subhankar et al. (2020), Zhao et al. (2022), and Howell et al. (2024) excel in delineating features but lack contextual interpretability. Meanwhile, the single-point localization approach in Lucassen et al. (2023) effectively detects discrete B-lines but faces challenges with merged or closely spaced artifacts. These findings emphasize the need for further development of advanced, interpretable, and clinically adaptable models to address the complexities of B-line detection and localization in LUS.

## 3 Methods

### 3.1 Data collection and preprocessing

The data used in this study comprise a publicly available dataset and a locally curated dataset, given the name of the Mulago and Kiruddu Lung Ultrasound Dataset (M-K LUS), of patients with lung disease. Both datasets were devoid of any patient-identifiable information, ensuring compliance with privacy regulations and preserving anonymity throughout the research process.

#### 3.1.1 Public dataset

**Figure 2:**
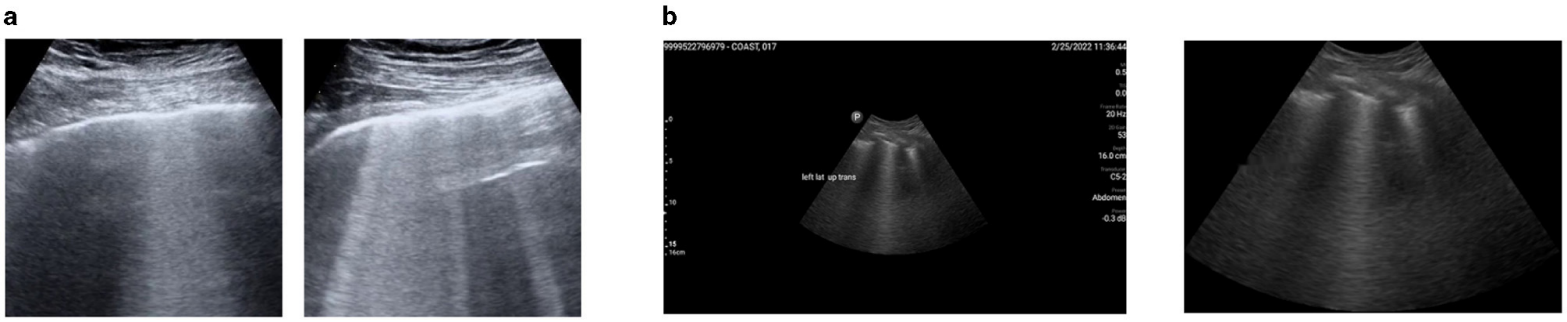
A representation of preprocessed LUS image frames from public and M-K LUS datasets. **(a)** Public dataset. Examples of image frames containing B-lines, extracted from LUS video data using a Python program. **(b)** M-K LUS dataset. Left: image frame with noise (ultrasound measure scales and text labels) before cropping. Right: corresponding image frame after cropping and the noise removed.

We downloaded 113 LUS videos from https://github.com/nrc-cnrc/COVID-US to https://github.com/jannisborn/covid19_pocus_ultrasound and selected 88 videos captured with a convex ultrasound probe. We focused on these LUS videos because our locally curated data included image frames obtained using the same probe type. Next, we divided the video data into training and validation sets at the patient level before extracting image frames, ensuring that all frames from a single video were assigned to the same set. Using a Python program, we extracted image frames at a rate of 5 Hz, yielding an average of 10 ± 4 frames per video, and a total of 926 images. In consultation with radiologists, we selected 212 unique images (170 for training and 42 for validation) containing B-line artifacts for annotation. Figure 2a shows a sample of image frames with B-lines preprocessed from a public dataset.

#### 3.1.2 M-K LUS dataset

Three senior radiologists, each with more than 10 years of experience in LUS, collected B-mode LUS images from patients suspected of COVID-19 or other pulmonary diseases. The patients were enrolled in a clinical study conducted at Mulago and Kiruddu referral hospitals in Uganda. The study obtained ethical approval from the Mulago Hospital Institutional Review Board and the Uganda National Council for Science and Technology. The enrolled patients provided written consent, and the radiologists conducted bedside LUS scans on them. All lung ultrasound scans were performed using Clarius C3 curvilinear (Clarius Mobile Health Corp, Burnaby, BC) and Philips Lumify C5-2 curvilinear (Philips Ultrasound, Bothell, WA, USA) handheld ultrasound probes, at frame rates between 15 and 30 frames per second using a low frequency (2–6 MHz) with preset configurations for depth, focal point, and gain. During the lung ultrasound examination, radiologists zoned every patient's thorax into 12 regions (left: upper posterior, lower posterior, upper anterior, lower anterior, upper lateral, lower lateral and right: upper posterior, lower posterior, upper anterior, lower anterior, upper lateral, lower lateral) and performed longitudinal and transverse scans on each region. Zoning the patient's thorax into 12 distinct regions during lung ultrasound examinations was essential for thorough and systematic assessment (Marini et al., 2021). It allowed the radiologists to comprehensively evaluate each part of the lungs and detect localized abnormalities, such as B-lines, consolidations, or pleural irregularities, which could be present in different lung zones (Duggan et al., 2020). Dividing the thorax into specific sections; upper and lower, anterior, posterior, and lateral on both the left and right sides, allowed radiologists to effectively identify the distribution and severity of lung involvement (Hernandez-Morgan et al., 2022). Additionally, longitudinally and transversely scanning within each region ensures that the examination captures different perspectives, improving the detection of hidden pathologies that might be missed with only one type of scan (Duggan et al., 2020).

The radiologist obtained 24 LUS image frames from each patient, stored them in a folder with the respective patient ID, and uploaded the folder to our local data server, which was created for this study. In the preprocessing phase, we excluded image frames without B-lines from each patient's folder using metadata provided by radiologists. We uniquely divided the folders into training, validation, and test sets, ensuring that image frames from the same patient were included in only one of the sets. Some images were of low quality, while others included noise in the form of LUS textual information. Poor-quality images were identified and removed in consultation with radiologists, and a noise reduction algorithm was applied to address the noise in the remaining 458 images (286 for training, 72 for validation and 100 for testing). The algorithm initially crops the image using the OpenCV library and then utilizes the keras_ocr library to detect text within the images. The identified text is subsequently in-painted using a Python function. Figure 2b shows an image frame with noise before cropping, alongside the same frame after being cropped and processed with the noise reduction technique integrated into the model.

### 3.2 Data annotation

**Figure 3:**
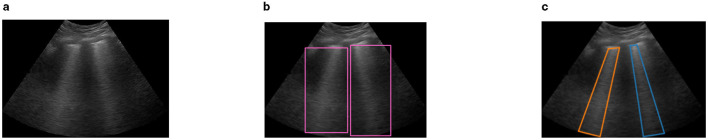
Sample image frame illustrating B-line artifacts and their corresponding annotations. **(a)** Original LUS image frame showing B-line artifacts. **(b)** RBB annotation highlighting the B-line region. **(c)** PBB annotation providing a precise contour of the B-lines.

We used the Visual Geometry Group Image Annotator (VIA) to annotate both the publicly available dataset and the M-K LUS dataset for training and validating the deep learning model. The VIA is a highly efficient web-based tool renowned for its intuitive interface and powerful image labeling features (Dutta and Zisserman, 2019). We began the annotation process by launching the VIA tool and uploading the images. In consensus with the radiologists, we applied two types of annotations to each image: RBB and polygonal bounding box (PBB), as illustrated in Figure 3. The RBB annotation provided a straightforward, encompassing outline for the B-lines (Figure 3b), while the PBB allowed for more precise, contour-specific labeling (Figure 3c). This dual approach in annotation was aimed at capturing the variability in the shape and orientation of B-lines, enabling the model to learn comprehensive localization strategies.

### 3.3 Proposed architectures

This work suggests a model based on the YOLO network structure for the localization of B-lines. At the core of YOLO's architecture lies the CNN, a specialized neural network that processes grid-like data, such as images. CNNs are adept at extracting spatial hierarchies from image data through convolutional layers, which apply filters to identify features such as edges, textures, and shapes. These features are then propagated through successive layers, enabling YOLO to comprehensively represent the image. The mathematical representation of a basic convolutional layer can be expressed as:


$$Y\left(i,j\right)=\sigma \left(\underset{m}{\sum }\underset{n}{\sum }X\left(i+m,j+n\right)\cdot K\left(m,n\right)+b\right)$$

Where:

- *Y*(*i, j*) represents the output of the convolutional layer at position (*i, j*)
- σ is the activation function
- *X*(*i*+*m, j*+*n*) corresponds to the input data
- *K*(*m, n*) denotes the convolutional kernel
- *b* is the bias term

YOLO divides an image into a grid and assigns responsibility for detecting objects to specific grid cells. Each cell predicts a fixed number of bounding boxes, confidence scores and class probabilities. The direct prediction approach eliminates the need for complex pipelines and region proposals, common in earlier detection models like R-CNN and Fast R-CNN. This streamlined process allows YOLO to achieve real-time detection performance while maintaining competitive accuracy. The mathematical formulation for calculating bounding box coordinates (x, y, w, h) and class probabilities (Pc) can be expressed as:


$$\begin{array}{l} B_{x}=\sigma (T_{x})+c_{x}\\ B_{y}=\sigma (T_{y})+c_{y}\\ B_{w}=p_{w}\cdot e^{t_{w}}\\ B_{h}=p_{h}\cdot e^{t_{h}}\\ \;P_{c}=\sigma (t_{c}) \end{array}$$

Where:

- *B*_*x*_ and *B*_*y*_ represent the predicted center of the bounding box
- *B*_*w*_ and *B*_*h*_ represent the width and height of the bounding box
- *P*_*c*_ is the confidence score
- σ is the logistic sigmoid function
- *T*_*x*_, *T*_*y*_, *t*_*w*_, *t*_*h*_, *t*_*c*_, *c*_*x*_, and *c*_*y*_ are the predicted parameters

In this study, we propose two architectures: YOLOv5-PBB and YOLOv8-PBB.

#### 3.3.1 YOLOv5-PBB

We developed YOLOv5-PBB to precisely localize B-lines in LUS images by modifying the YOLOv5 architecture, an object detection framework designed for RBB localization (Jocher et al., 2021). The YOLOv5 architecture consists of a backbone network, a neck network, and a prediction output head. The backbone network extracts features from input images at multiple scales using convolutional operations. The neck network merges these features, typically employing a feature pyramid structure to combine low-level features with high-level, abstract representations. Finally, the prediction head generates the final outputs, including object locations (bounding boxes), classes, and confidence scores. To develop YOLOv5-PBB, we made the following changes at multiple levels in the model's architecture and codebase.

**Figure 4:**
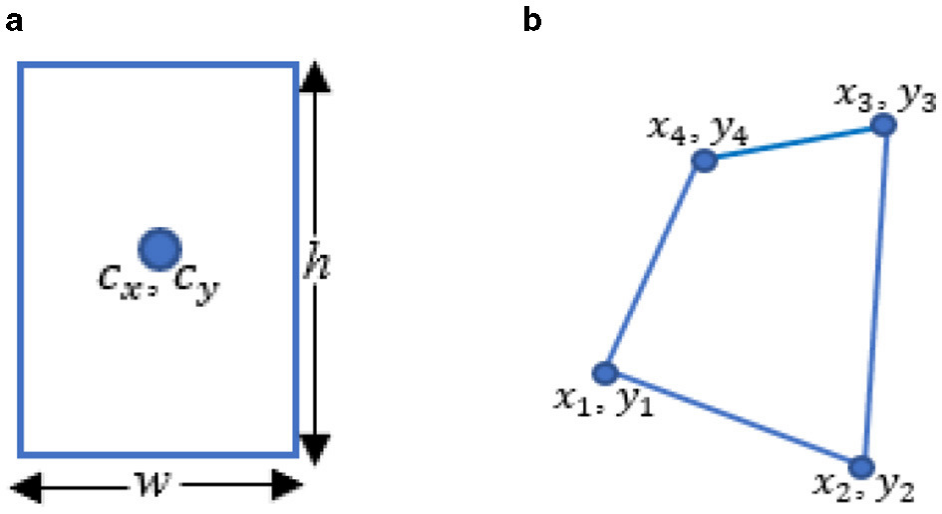
Comparison of detection head outputs of YOLOv5 and YOLOv5-PBB. **(a)** The RBB output of YOLOv5 includes *c*_*x*_ and *c*_*y*_, which represent the center coordinates of the RBB, while *w* and *h* indicate its width and height. **(b)** The PBB output of YOLOv5-PBB consists of *x*_1_, *y*_1_, *x*_2_, *y*_2_, *x*_3_, *y*_3_, and *x*_4_, *y*_4_, which represent the coordinates of the corners.

i) Converted the RBB output format (*c*_*x*_, *c*_*y*_, *w*, *h*) (Figure 4a) of YOLOv5 to the PBB format (*x*_1_, *y*_1_, *x*_2_, *y*_2_, *x*_3_, *y*_3_, *x*_4_, *y*_4_) (Figure 4b). Here, *c*_*x*_ and *c*_*y*_ represent the center coordinates of the RBB, while *w* and *h* denote its width and height. The PBB is defined by its corner coordinates, subject to the following constraints: *y*_3_, *y*_4_≥*y*_1_, *y*_2_; *x*_1_ ≤ *x*_2_; and *x*_4_ ≤ *x*_3_. Figure 4 illustrates a comparison between the RBB format used in YOLOv5 and the PBB format introduced in YOLOv5-PBB.
ii) Modified the Intersection over Union (IoU) and Non-Maximum Suppression (NMS) computations to work with PBB instead of rectangular ones. We used a custom function to compute the IoU for overlapping polygons. The function uses *shapely*, a Python library, to compute the intersection area and the union area to derive IoU. For the NMS, we replaced torchvision.ops.nms, a function in the Torchvision library designed to perform NMS on RBBs, with polygon-aware kernels to adjust the prediction tensor structure, bounding box representation, and confidence calculations.
iii) Enhanced the compute loss functions for RBB detection to support loss computation for PBBs. Specifically, we implemented constraints on the order of polygon vertices to ensure a consistent arrangement and utilized Smooth L1 loss for polygons to address vertex misalignment. Additionally, we created a custom function to match anchors with targets by computing the minimum bounding rectangle of polygons and considering shape-based heuristics (width and height of the bounding box enclosing the polygon).
iv) Developed custom data loader functions to process annotations and perform augmentations for polygonal datasets. Specifically, we designed a logic to manage polygon-specific data structures and transformations for annotations and utilized Albumentations to perform polygon-specific augmentations.

#### 3.3.2 YOLOv8-PBB

We developed YOLOv8-PBB from the YOLOv8 segmentation model, utilizing its segmentation capabilities to generate object masks, which are then post-processed to approximate polygon shapes by fitting polygons to the segmented masks. Instead of masking the B-line artifacts, the YOLO-PBB generates PBBs using a custom post-processing Algorithm 1, preserving the fine-grained details of the artifacts. To achieve this, we implemented the following:

i) Developed Algorithm 1 for detecting the contours of B-line artifacts in the segmentation mask and approximating them as polygons. These contours typically consist of many points representing the B-line's boundary. The polygonal approximation reduces the number of points based on a simplification criterion defined by ϵ, ϵ = 0.02 × arcLength(contour, True) as illustrated in Algorithm 1. The exact number of points for each polygon depends on the shape and complexity of the contours in the segmentation mask.
ii) Integrated Algorithm 1 into the post-processing function of the YOLOv8 segmentation model.

**Algorithm 1:**
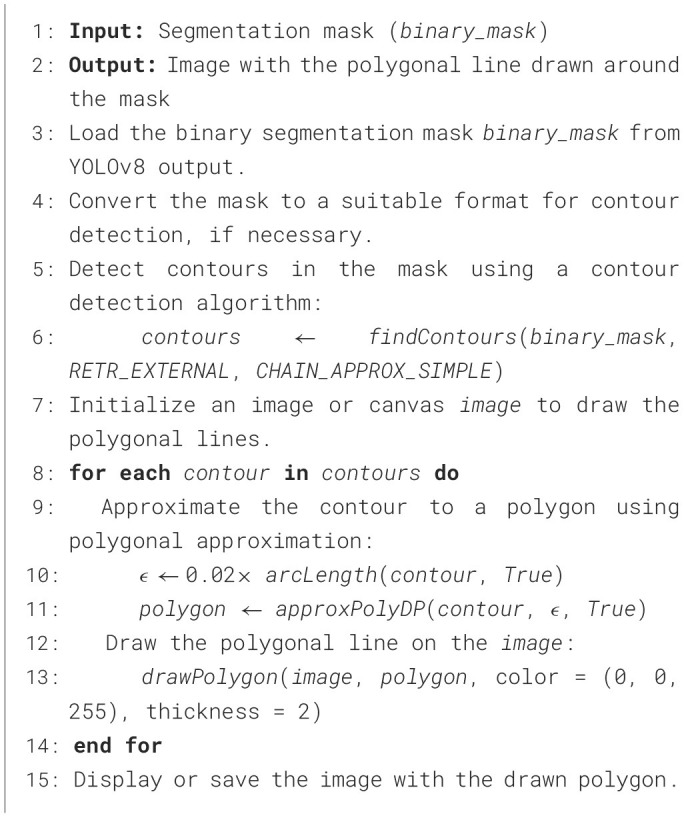
Contour detection algorithm for generating PBBs around B-line segmentation masks.

### 3.4 Model cost function

The cost (loss) function of YOLOv5-PBB comprises of:

- Bounding box loss (*L*_*box*_): measures the difference between predicted polygon coordinates and ground truth.
- Objectness loss (*L*_*obj*_): measures how well the model predicts whether the B-line object is present in a grid cell.

To compute the *L*_*box*_, the following are computed:

- CIoU loss (*L*_*CIoU*_): a metric that considers the overlap and geometric distance between predicted and target polygons, defined as;

$$L_{CIoU}=1-IoU\left(p_{box},t_{box}\right)$$

where *IoU*(*p*_*box*_, *t*_*box*_) is the intersection over union for the predicted polygonal bounding box (*p*_*box*_) and the ground truth polygonal bounding box (*t*_*box*_).

- Vertex ordering constraints (*L*_*order*_): ensures the predicted polygon maintains correct geometric constraints. It is defined as;

$$L_{order}=\frac{1}{6}\overset{6}{\underset{i=1}{\sum }}\max{\left(0,p_{i}-p_{j}\right)}^{2}$$

where *p*_*i*_ and *p*_*j*_ are pairs of vertices ensuring correct ordering.

- SmoothL1 loss (*L*_*vertex*_): penalizes errors in polygon vertex predictions. It is defined as;

$$L_{vertex}=\text{SmoothL1}\left(p_{box},t_{box},\beta =0.11\right)$$

The final bounding box loss is,


$$L_{box}=L_{CIoU}+L_{order}+L_{vertex}$$

The *L*_*obj*_ is computed using Binary Cross-Entropy (BCE) as;


$$L_{obj}=\overset{N}{\underset{i=1}{\sum }}BCE\left(p_{obj}^{\left(i\right)},t_{obj}^{\left(i\right)}\right)$$

where, $p_{obj}^{\left(i\right)}$ is the predicted objectness score and $t_{obj}^{\left(i\right)}$ is the target objectness score.The total loss is given by:


$$L=\lambda_{box}L_{box}+\lambda_{obj}L_{obj}$$

where λ_*box*_, λ_*obj*_ are hyperparameters controlling the importance of each term. The loss function weights are optimized using two approaches:

i) Static weighting via predefined hyperparameters

$$\begin{array}{l} \text{lbox}\;*=\text{self}\;.\;\text{hyp}\;[’\;\text{box}\;’]\\ \text{lobj}\;*=\text{self}\;.\;\text{hyp}\;[’\;\text{obj}\;’] \end{array}$$

ii) Dynamic weighting for the lobj when autobalance=True.

The total loss of YOLOv8-PBB is computed as the sum of several individual losses:


$$L_{total}=\lambda_{1}L_{obj}+\lambda_{2}L_{CIoU}+\lambda_{3}L_{dfl}+\lambda_{4}L_{seg}$$

λ_1_, λ_2_, λ_3_, λ_4_ are weighting factors to balance the contributions of the individual losses.*L*_*dfl*_ is the distribution focal loss, which focuses on learning the confidence scores for B-line detection, defined as;


$$L_{dfl}=\frac{1}{N}\overset{N}{\underset{i=1}{\sum }}\left[L_{\text{CE}}\left(p_{i},t_{l}\right)\cdot w_{l}+L_{\text{CE}}\left(p_{i},t_{r}\right)\cdot w_{r}\right]$$

where:

- *t*_*l*_ and *t*_*r*_ are left and right targets, respectively
- *L*_CE_(*p*_*i*_, *t*) is the cross-entropy loss for the predicted probability *p*_*i*_ and the target *t*,
- *N* is the total number of samples,
- *p*_*i*_ is the predicted probability for the *i*-th element in the distribution.
- *w*_*l*_ and *w*_*r*_ are the weights assigned to the left and right targets, respectively

*L*_*seg*_ is the segmentation mask loss, typically computed as a BCE loss for the predicted segmentation masks compared to the ground truth masks.


$$L_{seg}=\frac{1}{\text{area}}\underset{i}{\sum }BCE\left({\hat{M}}_{i},M_{{\text{gt}}_{i}}\right)$$

where ${\hat{M}}_{i}$ and *M*_gt_*i*__ are the predicted and ground truth masks for the *i*-th anchor, and area_*i*_ is the area of the corresponding bounding box.

The loss optimization process for YOLOv8-PBB involves calculating individual losses, summing and weighting them, using backpropagation to compute gradients, updating the model's weights with an Adam optimizer, and repeating this process until convergence.

### 3.5 Model training and parameter configuration

We conducted experiments on a system running Windows 10, utilizing PyTorch 2.5 as the deep learning framework and Python 3.10 for the programming environment. We trained YOLOv5 and YOLOv8 to localize B-lines using RBB, YOLOv8-OBB to localize B-lines using the oriented bounding box (OBB), YOLOv8-SEG to segment B-lines, and YOLOv5-PBB and YOLOv8-PBB to localize B-lines using PBB in LUS images. We trained and validated the models using the public dataset, the M-K LUS dataset, and a mixed dataset that combined the public and M-K LUS datasets.

During training, we optimized the models using the AdamW optimizer, which integrates the Adam algorithm with weight decay regularization to improve generalization. We configured the optimizer with an initial learning rate of 0.01, a momentum value of 0.937, and a weight decay coefficient of 0.0005. We resized input LUS images to 320 × 320 pixels and applied horizontal flipping and mosaic data augmentations by adjusting their parameters in the model training pipeline. Horizontal flipping was applied with a 50% probability, meaning each image had a 50% chance of being flipped horizontally during training. This technique helps the model learn spatial invariance to left-right orientation changes. The mosaic augmentation, with a scaling factor of 1.0, combines four images into one, offering diverse contextual information and enabling the model to detect and recognize features from different perspectives. We set the batch size to 16 and trained the models for 500 epochs, providing sufficient time for them to learn and converge on the relevant features.

### 3.6 Model evaluation metrics

We used precision (P), recall (R), and mean average precision (MAP) metrics to evaluate the models.

- P: Measures the proportion of true positive detections among all the instances identified by the model as positive. It is calculated as the ratio of true positives to the sum of true and false positives, indicating the model's ability to avoid false detections.
- R: Also known as sensitivity, quantifies the model's ability to identify all true positive instances correctly. It is calculated as the ratio of true positives to the sum of true positives and false negatives, reflecting the model's ability to detect all relevant B-line artifacts in the ultrasound images.
- mAP: Used to assess the overall performance of the model across different detection thresholds. It is the mean of the average precision scores for each class, providing a comprehensive measure of both precision and recall across various conditions. This metric is particularly useful for evaluating object localization tasks where precise detection and correct classification are critical. Higher mAP values indicate greater prediction accuracy.

## 4 Results

### 4.1 mAP and bounding box loss trends of the models

**Figure 5:**
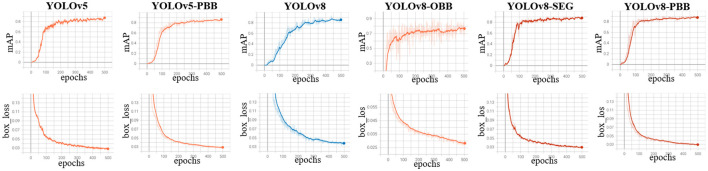
Training curves for YOLOv5, YOLOv5-PBB, YOLOv8, YOLOv8-OBB YOLOv8-SEG, and YOLOv8-PBB models when trained on the mixed dataset. The top row shows the mean average precision curves, while the bottom row depicts the box loss curves of the corresponding model.

We monitored the mAP and bounding box loss of the models to evaluate their performance during training on the mixed dataset over 500 epochs. Figure 5 presents the mAP and bounding box loss curves for all models, showing a rapid rise in mAP during the initial epochs before stabilizing. In contrast, the box loss steadily declines and converges to a lower value as training progresses.

### 4.2 Precision, recall and mAP values of the models

We evaluated YOLOv5, YOLOv5-PBB, YOLOv8, YOLOv8-OBB, YOLOv8-SEG and YOLOv8-PBB models on a held-out test dataset derived from the M-K LUS dataset following training on public, M-K LUS and mixed datasets. The evaluation results, including the P, R, and mAP values for each model, are presented in Table 1. It is observed that both models attained the lowest values of P, R and mAP when trained on a public dataset and the highest when trained on a mixed dataset.

**Table 1:** The P, R, and mAP values of YOLOv5, YOLOv5-PBB, YOLOv8, YOLOv8-OBB, YOLOv8-SEG and YOLOv8-PBB models trained on public, M-K LUS, and mixed datasets, and evaluated on a held-out test dataset derived from the M-K LUS dataset.

**Training dataset**	**Model**	**P**	**R**	**mAP**
Public	YOLOv5	0.862	0.835	0.863
	YOLOv5-PBB	0.895	0.871	0.892
	YOLOv8	0.872	0.855	0.887
	YOLOv8-OBB	0.890	0.862	0.892
	YOLOv8-SEG	0.898	0.862	0.893
	YOLOv8-PBB	**0.901**	**0.886**	**0.897**
M-K LUS	YOLOv5	0.867	0.843	0.896
	YOLOv5-PBB	0.920	0.911	0.933
	YOLOv8	0.897	0.878	0.901
	YOLOv8-OBB	0.900	0.855	0.904
	YOLOv8-SEG	0.910	0.909	0.928
	YOLOv8-PBB	**0.938**	**0.917**	**0.954**
Mixed	YOLOv5	0.891	0.886	0.898
	YOLOv5-PBB	0.931	0.918	0.936
	YOLOv8	0.913	0.902	0.920
	YOLOv8-OBB	0.926	0.906	0.928
	YOLOv8-SEG	0.929	0.913	0.930
	YOLOv8-PBB	**0.947**	**0.926**	**0.957**

Bold values indicate the highest performance in each dataset category.

### 4.3 Qualitative results of the models

**Figure 6:**
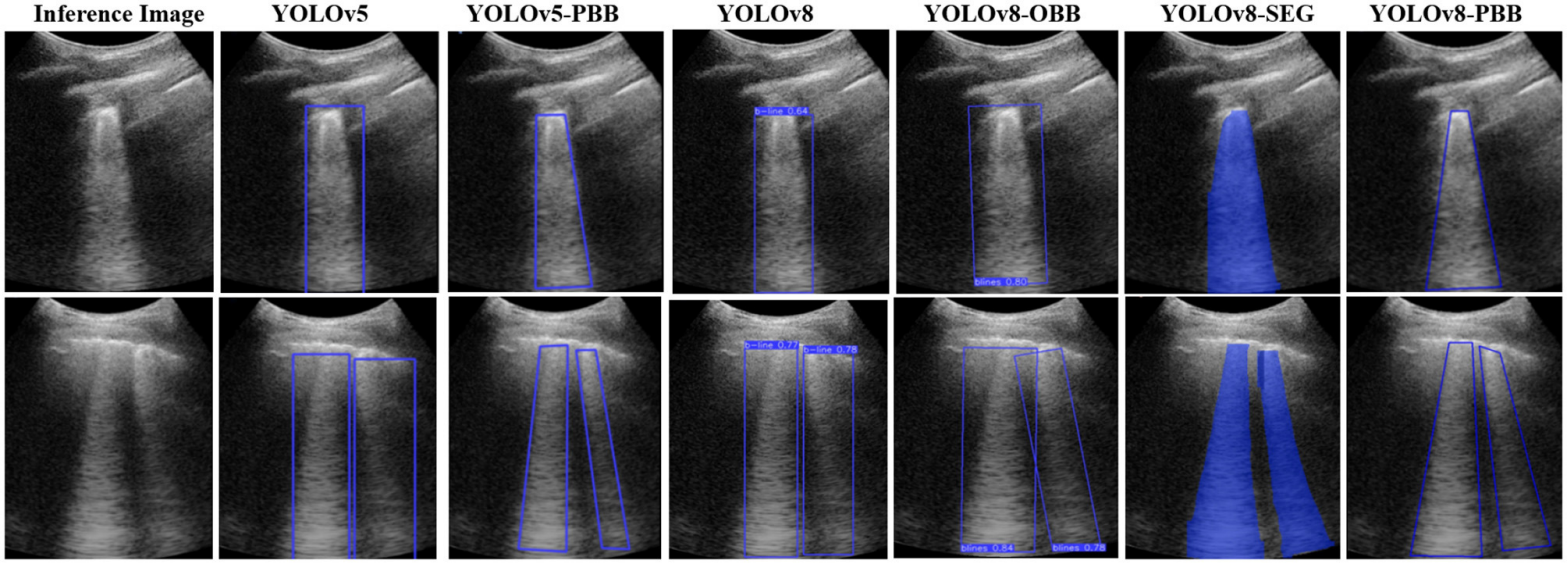
Comparison of B-line localization by YOLOv5, YOLOv5-PBB, YOLOv8, YOLOv8-OBB, YOLOv8-SEG, and YOLOv8-PBB models on two sample inference images from the held-out test set.

Figure 6 illustrates the qualitative results for B-line localization using the YOLOv5, YOLOv5-PBB, YOLOv8, YOLOv8-OBB, YOLOv8-SEG, and YOLOv8-PBB models, providing valuable insights into the interpretability of these models. The results pertain to two sample images from the held-out test set, each model trained on the mixed dataset. It is observed that B-line localization with PBB captures the B-line shape more accurately compared to RBB and OBB localizations, while segmentation using YOLOv8-SEG lacks contextual interpretability.

## 5 Integration of the model into a mobile LUS screening tool

### 5.1 Model integration

**Figure 7:**
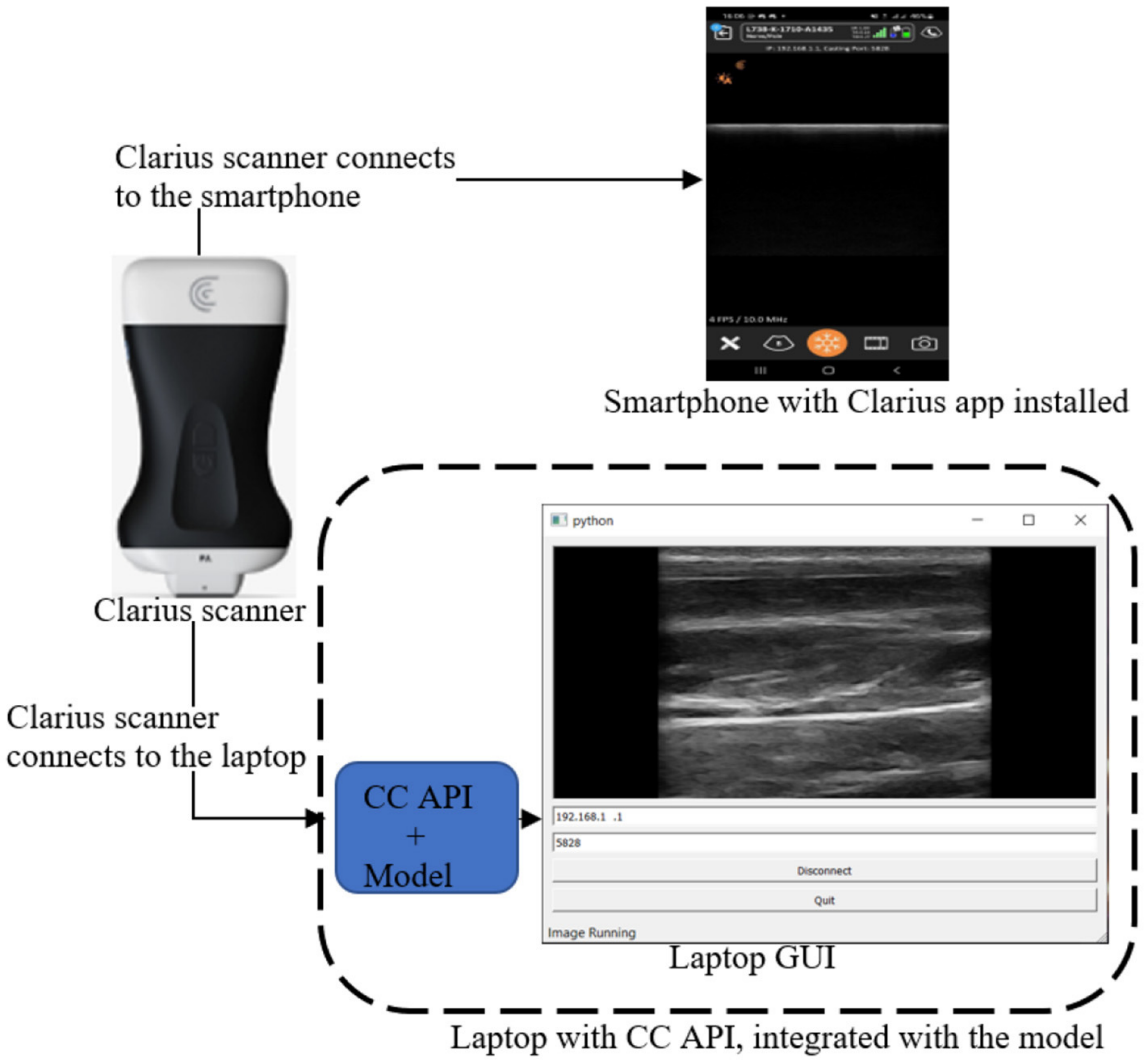
Mobile LUS screening tool comprising a Clarius scanner, a smartphone running the Clarius app, and a laptop equipped with both the CC API the model.

The mobile LUS screening tool consists of a portable ultrasound Clarius scanner, a smartphone equipped with the Clarius ultrasound application (Clarius app), and a laptop equipped with both the Clarius Cast application programming interface (CC API) and the model, as shown in Figure 7.

The integration process began with downloading the Clarius app from the Google Play Store and installing it on the smartphone. Next, we downloaded the CC API binary files (version 9.1) along with the supporting libraries (cast.dll, cast.lib, pycaster.py, and pysidecaster.py) from https://github.com/clariusdev/cast. Finally, we modified the pysidecaster.py script to incorporate the model and convert image frames into QuickTime (Qt) format.

During operation, the Clarius scanner initially connects to both the smartphone and the laptop, as shown in Figure 7. Once the connections are established, the user examines the patient with the scanner, which streams the scanned image frames simultaneously to the smartphone and the laptop. On the laptop, the model processes the image frames, detecting and localizing B-lines using PBB. Finally, the image frames are converted to Qt format and displayed on the laptop screen.

### 5.2 Model inference times

We conducted inference on test data using both YOLOv5-PBB and YOLOv8-PBB, measuring their inference times both before integration into the mobile LUS screening tool (*T*_before_) and after integration (*T*_after_). Table 2 presents *T*_before_ and *T*_after_ for the two models on sample 30 images.

**Table 2:** Inference times (in milliseconds) for YOLOv5-PBB and YOLOv8-PBB on various images, measured before (*T*_*before*_) and after (*T*_*after*_) integration.

**Image file**	**Image size**	**YOLOv5-PBB**		**YOLOv8-PBB**	
		*T*_before_	*T*_after_	*T*_before_	*T*_after_
*bl*_100.*jpg*	320 × 256	9.0	28.8	9.2	44.0
*bl*_103.*jpg*	320 × 256	9.1	33.0	8.6	42.0
*bl*_115.*jpg*	320 × 256	8.8	29.0	8.5	45.8
*bl*_117.*png*	320 × 288	14.3	39.3	48.1	69.9
*bl*_118.*png*	320 × 256	9.8	34.4	9.2	42.4
*bl*_121.*png*	230 × 256	8.7	26.6	8.5	44.0
*bl*_123.*jpg*	230 × 256	8.8	29.0	8.6	42.2
*bl*_125.*png*	230 × 256	9.2	31.2	13.4	44.0
*bl*_128.*png*	288 × 320	15.2	40.4	47.1	66.0
*bl*_129.*jpg*	230 × 256	8.9	28.4	9.3	44.2
*bl*_14.*png*	256 × 320	10.0	33.1	9.5	42.0
*bl*_15.*png*	256 × 320	9.1	31.6	8.6	43.0
*bl*_16.*png*	320 × 288	10.1	34.0	10.3	47.0
*bl*_19.*png*	320 × 256	8.9	27.8	10.5	43.6
*bl*_21.*png*	224 × 320	8.9	27.6	8.7	41.7
*bl*_24.*png*	320 × 288	10.0	33.2	10.4	45.6
*bl*_26.*png*	256 × 320	9.0	32.0	9.4	42.9
*bl*_29.*png*	256 × 320	9.0	32.0	8.7	43.0
*bl*_3.*png*	256 × 320	9.0	31.7	8.7	43.0
*bl*_31.*png*	256 × 320	10.1	34.0	8.7	45.2
*bl*_36.*png*	256 × 320	8.9	29.4	8.7	42.6
*bl*_37.*png*	288 × 320	10.0	35.0	10.0	46.0
*bl*_39.*png*	288 × 320	10.0	35.1	9.9	46.6
*bl*_42.*png*	320 × 320	10.0	36.0	10.5	64.6
*bl*_43.*png*	320 × 320	10.0	32.2	9.3	51.3
*bl*_6.*png*	288 × 320	11.0	37.3	10.0	50.9
*bl*_60.*png*	320 × 288	12.0	38.8	10.6	53.0
*bl*_95.*png*	320 × 320	12.9	39.2	10.1	55.0
*bl*_97.*jpg*	320 × 256	10.2	36.2	9.3	50.1
*bl*_99.*jpg*	320 × 256	10.1	35.8	9.5	48.9
**Average**		**10.0**	**33.1**	**12.1**	**47.7**

Bold values indicate the highest performance in each dataset category.

Table 2 shows that the average inference time for YOLOv5-PBB increases from 10.0 ms before integration to 33.1 ms after integration, while for YOLOv8-PBB, it rises from 12.1 ms to 47.7 ms. The rise in average inference times for both models underscores the influence of hardware and environmental factors on inference performance. Before integration, the model ran on an optimized cloud-based infrastructure with high-performance GPUs, enabling faster processing. After integration, however, the model operates on a local machine with potentially lower hardware specifications (e.g., CPU capabilities), resulting in longer inference times. Additionally, it is observed that the inference times generally correlate with the image size. Larger images tend to require more processing time, which is reflected in the increases in time after integration.

Furthermore, Table 2 shows that YOLOv8-PBB consistently has higher inference times on all images compared to YOLOv5-PBB, both before and after integration. This could be attributed to the additional complexity of YOLOv8-PBB, including segmentation and extra post-processing steps, as well as its larger model size of 21 MB compared to 14 MB for YOLOv5-PBB.

## 6 Discussion

The performance of the five deep learning models, YOLOv5-RBB, YOLOv8-RBB, YOLOv8-OBB, YOLOv5-PBB, and YOLOv8-PBB, trained on three different datasets (public, local, and mixed) and evaluated on a held-out local test set, is summarized in Table 1. The results offer valuable insights into how dataset diversity influences model performance. Overall, all the models achieved higher P, R, and mAP values when trained on the mixed dataset, compared to the public or MK-LUS dataset. This enhanced performance is supported by Shea et al. (2023) and Joseph et al. (2023), who suggest that dataset diversity helps deep learning models learn more robust features and improves their ability to generalize to unseen data. Specifically, the YOLOv8-PBB attained the highest performance among all models, with *P* of 0.901, *R* of 0.886, and mAP of 0.897. In contrast, YOLOv5 recorded the lowest values, with *P* at 0.862, *R* at 0.835, and mAP at 0.863. These results are consistent with findings in prior studies that suggest that more recent YOLO variants, such as YOLOv8, benefit from enhanced feature extraction and localization capabilities (Khan et al., 2024).

The qualitative results in Figure 6 show that the YOLOv5-PBB and YOLOv8-PBB models generate precise polygonal bounding boxes that closely follow the shape of B-line artifacts. These findings align with Li et al. (2020), which demonstrated that polygonal bounding boxes are more effective at detecting irregularly shaped objects compared to traditional rectangular bounding boxes. In contrast, YOLOv5-RBB and YOLOv8-RBB did not tightly capture the polygonal structure of the B-line artifacts and included irrelevant regions. This is consistent with Yang et al. (2021), which highlighted that rectangular bounding boxes are less effective for detecting elongated objects.

In Table 2, YOLOv5-PBB demonstrated a lower average inference time than YOLOv8-PBB, both before and after integration into the mobile LUS screening tool. The larger model size of YOLOv8-PBB (21 MB vs. 14 MB for YOLOv5-PBB) contributed to greater model complexity, leading to increased inference time. This observation aligns with findings from Hu et al. (2021) to Zawish et al. (2024), which highlight that deep learning model size influences complexity, with smaller models generally achieving faster inference times. Zawish et al. (2024) further emphasizes that balancing model accuracy and complexity is essential when choosing a model for resource-constrained Internet of Things environments.

## 7 Conclusion

This study modified YOLOv5 and YOLOv8 models into YOLOv5-PBB and YOLOv8-PBB, respectively, for detecting and localizing B-line artifacts in LUS images using polygonal bounding boxes (PBBs). The YOLOv5 adaptation involved modifying the detection head, loss function, non-maximum suppression function, and data loader, while YOLOv8 was enhanced with a custom post-processing algorithm to enable PBB-based localization. Additionally, a tailored image preprocessing technique was integrated to improve LUS image quality. Comparatively, YOLOv8-PBB achieved slightly higher precision, recall, and mAP than YOLOv5-PBB, but YOLOv5-PBB was more lightweight and had a shorter inference time. Furthermore, each model was incorporated into a mobile LUS screening tool for utilization in resource-constrained settings with scarce expert radiologists.

A limitation of this study is its exclusive focus on localizing B-line artifacts in LUS images. While the models effectively identified and counted isolated B-lines, they struggled in more complex scenarios where B-lines clustered together or merged into a continuous white line on the ultrasound image, making precise separation and counting difficult.

In the future, we plan to focus on the spatiotemporal localization of B-line artifacts and extend our work to include the detection of other artifacts, such as irregular pleural lines and consolidations, at both the frame and video levels. Additionally, we aim to explore semi-supervised learning techniques to reduce annotation efforts and improve scalability. Furthermore, we intend to evaluate the model on multi-center datasets from diverse geographic regions to improve generalizability.

## Data Availability

The raw data supporting the conclusions of this article will be made available by the authors, without undue reservation.
